# Welcoming new neighbors: Minnesota's rapid response model to address the urgent health needs of Afghan newcomers, 2021–2022

**DOI:** 10.3389/fpubh.2024.1413258

**Published:** 2024-06-26

**Authors:** Mateo Frumholtz, William C. Carlson, Patricia J. Shannon, Sophia Iaquinta, Maggie Eckerstorfer, Brett Hendel-Paterson, Nasreen Quadri, Rashika Shetty, Hadia Mohammadzadah, William Stauffer, Opeyemi Adesida, Cindy Howard, Kailey Urban, Jonathan Kirsch, Mehria Sayad, Blain Mamo

**Affiliations:** ^1^Minnesota Department of Health, Saint Paul, MN, United States; ^2^School of Social Work, University of Minnesota, Minneapolis, MN, United States; ^3^School of Nursing, University of Minnesota, Minneapolis, MN, United States; ^4^Department of Medicine, Global Medicine, University of Minnesota, Minneapolis, MN, United States; ^5^Mobile Health Initiative, University of Minnesota, Minneapolis, MN, United States; ^6^Internal Medicine, HealthPartners Institute, Minneapolis, MN, United States; ^7^National Resource Center for Refugees, Immigrants, and Migrants, University of Minnesota, Minneapolis, MN, United States; ^8^Department of Pediatrics, University of Minnesota, Minneapolis, MN, United States; ^9^Medical School, University of Minnesota, Minneapolis, MN, United States; ^10^Infectious Disease, HealthPartners Institute, Minneapolis, MN, United States; ^11^Community-University Health Care Center, University of Minnesota, Minneapolis, MN, United States

**Keywords:** Afghanistan, public health response, refugees, immigration, trauma-informed care, Operation Allies Welcome

## Abstract

As a result of the United States withdrawal from Afghanistan in fall 2021, 1,260 Afghan evacuees arrived in Minnesota between October 2021 and February 2022. Several contextual factors including an overtaxed health system under duress from COVID-19 and uncertain benefit eligibility prompted a coordinated public health response to appropriately address the acute and pressing medical concerns of our new neighbors. This community case study describes the State of Minnesota's cross-sectoral response that created a welcoming environment, identified public health concerns, and addressed acute medical needs. Medical volunteers provided an initial health and safety check for Afghan families upon arrival. Volunteers also offered onsite culturally and linguistically appropriate mental health assessments, group therapy, women's clinics, vaccine clinics, medication refills, and ongoing walk-in primary care. Care coordinators facilitated primary care and specialty care referrals. The majority (96%) of eligible arrivals were screened as part of this response and the median time between arrival to Minnesota and initial health screening was 2 days. Half of all arrivals screened reported at least one health concern and 56% were referred to a specialty for further evaluation. Almost one in four adults (24%) reported mental health concerns. Existing partnerships across local sectors can be leveraged to provide comprehensive physical and mental health services to newcomers in an emergency response.

## 1 Introduction

State public health departments increasingly collaborate with human services and other government agencies, local emergency resources, and higher education institutions to protect and address community wellbeing immediately following natural and man-made disasters (1). The fall of the Afghan government in August 2021 and the United States (U.S.) Department of State's subsequent evacuation of 84,600 Afghans over 17 days sparked an emergency response and resettlement pathway, organized through Operations Allies Welcome (OAW) (2). Although migration crises such as this are not unprecedented, the U.S. government's response certainly was atypical. Ongoing civil disruptions and climate change have exacerbated forced human displacement and novel case studies can provide meaningful guidance to improve future responses (3).

The literature describing medical responses during OAW is currently limited to military efforts that occurred in and around the domestic military bases called Safe Havens (4–9). Most evacuees initially spent 1–6 months at one of eight Safe Havens where they received basic medical care and vaccines prior to resettling across the U.S. (4). This community case study describes Minnesota's coordinated public health process model created to respond to the health needs of arriving Afghan families between October 2021 and February 2022. This unique response contributes to the literature related to public health collaborations across local, state, and university resources. Lessons learned from this case study can inform local planning to build resilient community health systems in the face of future emergencies.

## 2 Context

Relevant contextual factors that prompted the implementation of a rapid response team and drove decision-making include: (1) an emergent humanitarian crisis and atypical resettlement process that resulted in the omission of overseas medical evaluations, (2) acute medical and mental health needs, (3) an inadequate refugee resettlement infrastructure for meeting the complex needs of evacuees, (4) an overtaxed healthcare system responding to COVID-19, and (5) uncertain immigration status and limited access to services due to temporary humanitarian parole.

### 2.1 Emergent humanitarian crisis

The U.S. withdrew from Afghanistan in < 2 months after reaffirming the original declaration of U.S. military withdrawal by 2021 (2). This rapid withdrawal and subsequent government change exacerbated existing health insecurities in the wake of two decades under U.S. occupation (10). Traditional humanitarian migration processes involve lengthy refugee status applications, comprehensive vetting, acceptance for resettlement to a host country, and health evaluations. In contrast, during the chaos of the U.S. withdrawal, Afghan evacuees rapidly boarded U.S. military planes at the Kabul airport and were transported directly to the U.S. Safe Havens. In response to Afghan families' need for a resettlement pathway, the U.S. leveraged an existing temporary protection status, called Humanitarian Parole, available for emergency admissions to the U.S. (11). Congress then appropriated supplemental funds for Afghan families to receive federal refugee benefits and move out of Safe Havens via refugee resettlement programs across the U.S.

### 2.2 Acute medical and mental health needs exacerbated by trauma

Safe Havens shared limited medical information with receiving states. Early reports indicated that Afghan evacuees were presenting with acute medical illnesses, uncontrolled chronic medical conditions, complications of traumatic injuries, as well as infectious diseases such as measles, tuberculosis, polio, and Leishmaniasis (12). Early reports of the traumatic nature of evacuation processes prompted additional concerns about acute mental distress and the need for mental health services (13, 14).

Many Afghans experienced multiple traumatic events during evacuation including witnessing and experiencing threatened and actual violence, family separation, uncertain and unsafe waiting conditions, prolonged physical pain, and death (15). This acute trauma occurred in the context of an ongoing and historical war and political violence with many people having experienced personal trauma (16, 17). Prevalence studies indicated that 86% percent of Afghans have experienced at least one traumatic event (18) and that upwards of 80% of Afghan women report experiences with intimate partner violence (19). Exposure to these events has been shown to result in psychological distress for 47% of the Afghan population who have experienced at least one trauma (18).

### 2.3 Inadequate refugee resettlement infrastructure

Under the U.S. Refugee Admissions Program, resettlement agencies (RA) and local partners offer case management services to connect refugees to permanent housing, employment, educational opportunities, and healthcare services to complete a Domestic Medical Examination (DME; the initial health assessment for refugees and other newcomers in the U.S.). Per-capita federal funding and the agencies' private revenue sources support resettlement efforts. This private-public partnership model has provided consistent access to resettlement services for refugees arriving through this structured pathway (20).

Every October, the President of the United States authorizes the Presidential Determination on Refugee Admissions, establishing a ceiling for the maximum number of people with certain humanitarian visa statuses who can resettle in the country. During the Trump administration, this ceiling decreased from 85,000 in 2016 to 18,000 in 2021, the lowest number since the enactment of the Refugee Act of 1980 (21). RA funding and infrastructure paralleled the stark decrease in refugee admissions (22). Nationally, these funding cuts led to closures of local resettlement agencies, staffing shortages, and a weakened refugee resettlement infrastructure unable to respond to a surge of arrivals through OAW (23).

### 2.4 Overtaxed health care system

Minnesota Department of Health's Refugee Health Program (MDHRHP) consistently screens more than 95% of eligible arrivals with a DME through a network of participating clinics (24). These clinics link to larger primary care systems or public health clinics. Although refugees are usually connected to healthcare services within the first 90 days of resettlement, Minnesota's healthcare infrastructure did not have capacity to triage and respond to this sudden influx of Afghan families (210 arrivals per month on average, on top of other refugees arriving at the same time). Additionally, the ongoing toll of the COVID-19 pandemic severely limited clinic access, straining typical newcomer healthcare pathways. Given this context, the response team prioritized triaging Afghan families to facilitate efficient access to care.

### 2.5 Uncertain immigration status

Although the temporary Humanitarian Parole status granted evacuees limited initial resettlement services, the provision of health insurance remained uncertain. Delayed assurances from federal agencies and Congress regarding eligibility for federal benefits, such as health insurance, further motivated the need to provide preventative healthcare as early as possible. Regardless of eligibility for benefits offered by the Office of Refugee Resettlement (ORR), the Afghan Evacuee Health Response Team offered its services to all OAW arrivals in Minnesota.

## 3 Afghan health response process model

In response to these contextual challenges, a novel process was developed to meet emerging and anticipated needs of Afghan newcomers. The Minnesota Resettlement Programs Office (RPO) started the OAW Afghan Evacuee Response, an incident command system, to coordinate and implement timely wrap-around services for newcomers. Under the command of the RPO, MDHRHP was tasked to lead the health response operations. MDHRHP collaborated with the University of Minnesota's (UMN) Global Medicine and Global Pediatrics Programs, Mobile Health Initiative (MHI), and Medical Reserve Corps (MRC) to develop and implement an evidence-based and trauma-informed clinical protocol. This approach assessed physical and mental health needs through interdisciplinary collaboration to meet the complex needs of newcomers (25) and was informed by prior research on destigmatized and culturally acceptable service implementation (26).

The goals of this health response were to provide a warm welcome to arriving families, identify health conditions of public health concern, address acute and immediate ongoing medical and mental health needs, triage medical referrals to specialty systems, and facilitate establishment of ongoing primary care. Avoiding unnecessary urgent care and emergency department visits while providing best-practice refugee care (e.g., culturally and linguistically appropriate care by experienced refugee health providers) was a central objective.

The Arrival Health and Safety Checks (AHSC) were completed at the temporary housing facility between October 1, 2021, and February 17, 2022. During that time, MHI provided logistics support and overall onsite organization. MHI coordinated and deployed over 113 medical volunteers in 135 scheduled shifts onsite at the temporary housing facility totaling 1,317.5 volunteer hours over the five-month response.

Interdisciplinary volunteer teams included physicians, advanced practice providers, nurses, pharmacists, and health professions students or resident physicians, who were closely supervised by attending physicians. Providers represented family medicine, internal medicine, emergency medicine, pediatrics, obstetrics/gynecology, psychology, social work, and marriage and family therapy; most with experience addressing the physical and mental health of recently arrived refugee populations. When possible, gender concordance between patient and provider was prioritized. The MRC coordinated subsequent mental health referrals with a team of 18 onsite volunteer mental health specialists.

### 3.1 Development of the process model

This response focused on providing integrated healthcare to address the newcomers' immediate needs at a volunteer-run clinic in a temporary housing facility. The team addressed healthcare needs arising from the disaster event itself, including abrupt disruptions of long-term medical care, physical and psychological trauma associated with U.S. occupation and evacuation, and chronic medical conditions such as post-surgical care and medication refills.

The Arrival Health and Safety Check was developed using a standardized form based on the Centers for Disease Control and Prevention (CDC) newcomer health screening guidance and existing mental health screening tools (Supplemental material A). The Health and Safety Check included compiling existing medical and vaccination records from Afghanistan and Safe Havens, screening for diseases of public health significance, addressing acute health concerns, refilling medications, and screening for mental health support needs. The mental health team was intentionally referred to as the social support team in this model to be de-stigmatizing for Afghans and providers. A condensed version of the Minnesota Wellbeing and Emotions Check (*WE-Check*), a behavioral health assessment screening tool developed by MDHRHP, was used to identify individuals in need of referral to the MRC social support team for mental health assessment (27). The MRC social support team conducted follow-up assessments onsite to identify individuals in need of urgent, ongoing mental healthcare to address both acute trauma-related mental health needs and chronic mental health difficulties exacerbated by current and recent stressors. Care plans were developed for patients with complex mental and physical health needs to manage acute medical needs and expedite outpatient follow-up. The health services provided by this team evolved over the response period as we learned from Afghans about their needs and as initial service data were evaluated.

### 3.2 Description of the process model

**Figure 1:**
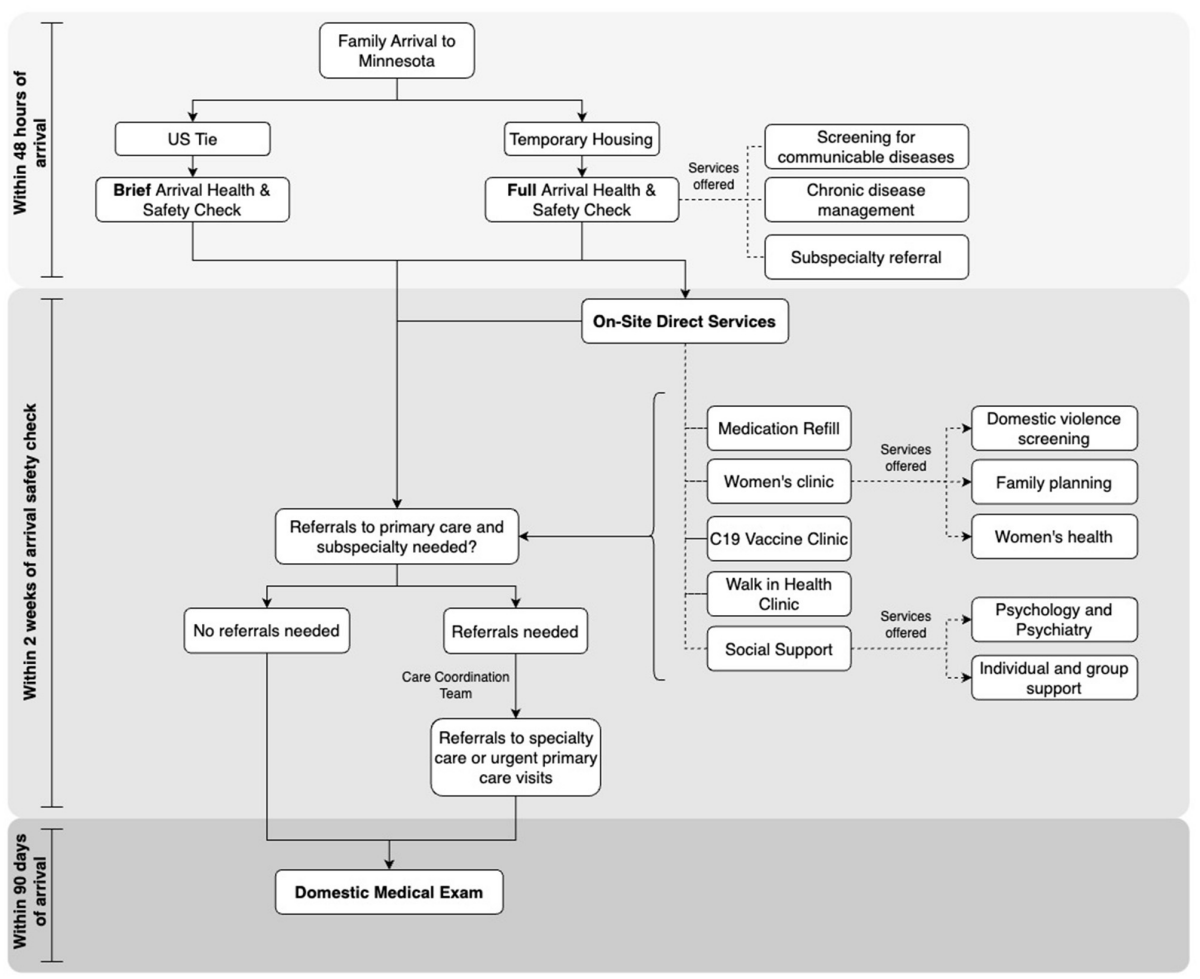
Process flow for afghan health and safety checks provided by the Afghan health response team.

Licensed medical providers, mental health specialists, and health advocates with experience in trauma-informed care collaborated to deliver the Arrival Health and Safety Checks and to triage individuals to additional onsite assessment and services, or offsite specialty referrals as needed. Leadership team members trained and supervised all volunteers. Figure 1 depicts the process flow of arriving families, health assessment, and subsequent connections to care as follows:

1. Upon notification of an upcoming arrival, RAs notified MDHRHP, who informed the MHI and MRC logistics teams about the timing of anticipated arrivals during a daily leadership and team meeting.
2. Afghan newcomers who stayed at a temporary housing facility completed the AHSC within 48 h of arrival. A limited number of people, who lived with family members in MN upon arrival received the full AHSC only if requested; otherwise, a case manager administered an abbreviated AHSC.
3. On-site direct services such as medication refills, Women's Clinic, COVID-19 vaccine clinic, walk-in health clinic, and mental health services were available to all newcomers as needed for the duration of the response.
4. After completion of the AHSC, a care coordination team scheduled specialty and urgent primary care follow-up appointments along with transportation and interpreters.
5. Families were integrated into the traditional connection-to-care model to complete their DME.

A system of arrival notification and processing of medical intake data was created using the HIPPA compliant REDCap software (Version 13.2.4). Providers submitted health assessment results on paper to MDHRHP who entered them into REDCap manually. R Studio (Version 4.3.1) was used for data processing and analysis.

### 3.3 Process model adaptations

The response consisted of four *phases* as the model evolved over time to accommodate environmental factors and meet the additional identified needs of newcomers. The *first phase* of the response aimed to reach newcomers at their point of entry, the Minneapolis-Saint Paul International Airport. MHI and MDHRHP collaborated with the emergency responders at the airport to establish a confidential space to set up operations. This phase consisted of greeting newcomers at the arrival gate and accompanying them to the health intake area, bringing in necessary medical equipment and allowing 24/7 access for volunteer providers who conducted the initial Arrival Health and Safety Checks. Within a few weeks, it became clear that this model was unsustainable given the unpredictability of flight schedules and increased volume of arrivals. Furthermore, preliminary analysis indicated low risk for communicable infections and that 24–48 h was sufficient for responding to emerging needs.

The *second phase* of the health response model moved the Arrival Health and Safety Check from the airport to the temporary housing facility. MHI equipped a large confidential room within the temporary housing facility to conduct Arrival Health and Safety Checks with the families. The medical and operations teams had a formulary of over-the-counter medications on-site and could dispense them as needed. A local pharmacy with a delivery service was also available to fill prescriptions and distribute directly to the temporary housing. This transition also allowed access to Dari and Pashto interpreters who provided services to the entire response operations. When available, interpreters with healthcare backgrounds were utilized.

The *third phase* involved the creation of the Women's Clinic. It was quickly noted that the presence of a large family during visits frequently minimized opportunities for women to discuss their health concerns. During the family Arrival Health and Safety Checks, all women 18 years and older were offered the opportunity to attend a follow-up appointment in the weekly Women's Clinic to discuss sensitive medical and social health concerns following best practices for gender- and culture-responsive care. These were identified through a rapid literature review, the Afghan American physician lead of the Afghan Health Response Team, and feedback from newly arrived Afghan women. Female medical providers and female interpreters staffed the Women's Clinic, ensuring a *women-serving-women* approach in a private and psychologically safe environment. Having female interpreters and providers was essential to enhance safety, increase comfort, and encourage discussion of mental health and medical concerns. For example, Afghan women shared their desire for access to contraception and family planning—a culturally private topic—which was difficult for many women to discuss openly in less-private mixed-gender settings. Evaluating female patients in the Women's Clinic, one provider noted: “It was like seeing a whole different person”.

Many women reported somatic symptoms of stress and anxiety. Weekly support groups provided women psychoeducation about acute stress reactions and self-care practices to minimize enduring stress responses. Group sessions included relaxation and mindfulness exercises, therapeutic yoga, and culturally acceptable social support with other women in the temporary housing site. MRC members with experience in children's mental health offered concurrent childcare with games and tailored activities to enable women to utilize these onsite supportive services. This dual-purpose resource also allowed newcomer children to build friendships through active play and interact positively with other adults at the temporary housing.

The *fourth phase* responded to the significant increase in single individuals and two person families, which prompted the opening of a secondary temporary housing site. MHI adapted the Arrival Health and Safety Check to fit within the nursing screening/triage scope of practice. Nurse volunteers provided the initial health assessment at the secondary housing facility and referred clients to medical providers at the nearby primary temporary housing, if needed. When Minnesota experienced a sharp increase in COVID-19 cases in early 2022, the MRC mental health appointments moved online to minimize the risk of spread within the temporary housing.

Another component of this response was the provision of secondary trauma support for the response staff and volunteers. The rapid pace of daily arrivals exacted a toll on volunteer providers who struggled to meet the daily demands while coping with learning about the traumatic nature of families' experiences. The leadership team also faced the daily stress of addressing emergent complex medical needs, preparing for new arrivals, and troubleshooting administrative challenges. An MRC leader provided secondary trauma support for leadership, medical and mental health volunteers, and all RA staff. The University of Minnesota's Center for Spirituality and Healing also offered sessions focused on stress management.

## 4 Results

Between October 1, 2021, and February 17, 2022, 1,260 Afghan evacuees across 386 families resettled to Minnesota as part of the OAW response. Over half (57%) were male and the median size of each family was 2 (range: 1-13). Forty-seven percent arrived as single individuals, of which 87% were male. The median age upon arrival to Minnesota was 18 years (range: 17 days−73 years) and 46% were 15 years of age or younger. Female arrivals were significantly younger than males with a median of 15.9 and 20.3 years, respectively. Most arrivals (85%) stayed at the temporary housing facility upon arrival and 29% arrived in January 2022 (Table 1).

**Table 1:** Demographics for OAW Afghan arrivals, Minnesota 2021–2022.

**Characteristic**	**Count**	**Percent**
**Gender**		
Female	527	42%
Male	733	58%
**Age**		
< 5	210	17%
5–11	261	21%
12–18	181	14%
19–30	317	25%
31–40	193	15%
41–50	66	5%
51–60	22	2%
61+	10	1%
**Month**		
October, 2021	131	10%
November, 2021	246	20%
December, 2021	249	20%
January, 2022	371	29%
February, 2022	263	21%
**Lodging**		
US Tie	195	15%
Temporary housing	1,065	85%
**Total**	1,260	

This data includes individuals arriving with any visa type (Legal Permanent Residents, Parolees, SIV, US Citizens, etc.) as part of OAW.

Of all arrivals, 1,206 (96%) people were screened with either a brief (*n* = 186) or full (*n* = 1,020) AHSC and 54 (4%) people were lost to follow-up. The brief AHSC was performed by RA's when families were directly resettling with U.S. family ties. Thus, data was only available for the 1,020 (81%) who completed the full Health and Safety Check. The median time between arrival to Minnesota and the initial Health and Safety Check was 2 days (range 0–41 days; IQR: 1–5 days). Median time to the Health and Safety Check increased over time: 0 days in October, 1 day in November, 2 days in December, and 4 days in January and February.

Half of all Afghan newcomers screened endorsed at least one health concern upon evaluation (Table 2). These included acute health conditions (23%), dental problems (17%), chronic conditions (14%), and vision problems (13%), among others (myalgia, dermatology, gastrointestinal, upper respiratory, fever, and family planning). Adults endorsed at least one health concern more often than children (67% vs. 33%). Most (56%) arrivals who were seen were referred to at least one healthcare specialty for further evaluation. Most arrivals over the age of 5 (90%) had received at least one COVID-19 vaccine at the Safe Havens, with 73% completing the primary COVID-19 vaccine series soon after resettling to Minnesota.

**Table 2:** Health concerns endorsed by Afghan arrivals, MN OAW 2021–2022.

	**Count (%)**		
**Health Concerns**	**Children**	**Adult (18**+**)**	**Overall**
Fever	9 (2%)	6 (1%)	15 (1%)
Upper respiratory	25 (5%)	11 (2%)	36 (4%)
Gastrointestinal	15 (3%)	51 (10%)	66 (6%)
Dermatology	29 (6%)	51 (10%)	80 (8%)
Myalgia	12 (2%)	100 (19%)	112 (11%)
Vision	28 (6%)	100 (19%)	128 (13%)
Chronic conditions	32 (6%)	115 (22%)	147 (14%)
Dental	37 (7%)	141 (27%)	178 (17%)
Acute health conditions	70 (14%)	168 (32%)	238 (23%)
At least one health concern^*^	164 (33%)	347 (67%)	511 (50%)

^*^At least one health concern includes endorsement of any health concern above except for the behavioral health questions (See Supplemental material A for more information).

This data includes individuals arriving with any visa type (Legal Permanent Residents, Parolees, SIV, US Citizens, etc.) as part of OAW.

Of the 212 women of reproductive age (15–49 years old) who completed a health check, 42 (20%) were pregnant upon arrival to Minnesota. Over a quarter (28%) of female newcomers were referred to an OBGYN specialist for pregnancy, gynecologic symptoms, or family planning. The largest proportion of internal referrals was for the Women's Clinic. More than half (51%) of women 18 and older were referred to the Women's Clinic and the majority (63%) attended.

When asked the *WE-Check* mental health screener questions, 124 (24%) adults endorsed being too sad in the past month, 139 (27%) endorsed worrying too much, and of those who endorsed at least one of the first two questions, 48 (28%) said these stressors made it difficult for them to take care of themselves and their families. Among those 18 or older, 123 (24%) were internally referred for mental health assessment (Table 3). Female arrivals were more often referred to the MRC social support team than males (17% and 12%, respectively). Arrivals 41–50 years old had the highest rate (38%) of social support team referral compared to any other age group. The median time between the Arrival Health and Safety Check and social support assessment was 6 days (range: 1–48 days). Two individuals with known mental health concerns upon arrival were assessed for social support before their Health and Safety Check.

**Table 3:** Specialty referrals after initial health and safety assessment, Minnesota 2021–2022.

**Specialty**	**No. referred**	Perc. referred (%)^*^	**% of referrals**
Mental health	123	24%	22%
Vision	100	10%	17%
Primary care	217	21%	38%
OBGYN	61	29%	11%
Dental	260	25%	45%
Pediatric	71	14%	12%
Women's clinic^**^	98	51%	17%
**Total Referrals**	572	56%	

^*^Percent referred of those screened and eligible for referral. Of 1,020 screened, 520 elligible for mental health (18+), 500 eligible for pediatric (< 18), 212 are women of reproductive age (15-49), and 193 are elligible for Women's clinic (18+).

^**^The first women's clinic was offered on December 9, 2021, although some women who arrived before were also referred.

This data includes individuals arriving with any visa type (Legal Permanent Residents, Parolees, SIV, US Citizens, etc.) as part of OAW.

## 5 Discussion

Migration-related humanitarian responses are likely to grow in the context of increasing climate-related crises, natural and man-made disasters, and geopolitical conflict (28, 29). This case study illustrates a creative approach to meeting the comprehensive health needs of acutely displaced Afghans arriving in the U.S. during a time of widespread health insecurity, resource scarcity, and systemic stress. Core lessons learned from the present case study include strategies to: (1) effectively mobilize local expertise in the context of chronic resource constraints, (2) develop integrated culturally responsive health assessment and care coordination that is responsive to the population, and (3) implement proactive and prompt interventions to address acute and chronic health needs.

### 5.1 Mobilizing local expertise to supplement resource constraints

Despite logistical challenges and delayed federal guidance and funding, MDHRHP relied on relationships with its local partners to respond to system gaps and limitations. MDHRHP has historically partnered with teams of global health care providers to provide trauma-informed cross-cultural assessment and treatment. Investment in these partnerships resulted in the ability to mobilize personnel and resources quickly. Leaders in these fields leveraged existing emergency response teams within local governments and the university to recruit and train additional professional volunteers. Local public health departments should consider building and sustaining relationships with community organizations and institutions of higher education that can be mobilized to assist with a disaster response. Consistent networking with established and budding community resources is an essential feature of systemic disaster preparedness. Through enduring collaboration driven by emerging and identified needs, this network of state, community, and university partners successfully met our goals of providing a safe and welcoming environment for Afghan families.

### 5.2 Integrated culturally responsive health assessment and care coordination

The MDHRHP oversaw an integrated approach that relied on the daily collaboration of co-leaders of medical, mental health, and case management response teams. This response structure created opportunities for creative problem-solving to improve the quality of care. Normalizing traumatic stress reactions and conversations about the interrelation of physical and mental wellbeing began during the newcomers' first meeting with a medical provider. The impact that these initial normalizing conversations had on newcomers was apparent during follow-up mental health assessments, brief interventions, and community referrals for newcomers in need of additional support. In addition to the medical team's initial support of mental health screening, the social support team's psychologists, social workers, and psychiatrists supported the work of the medical teams by responding to specialized situations involving traumatic losses, family separation, mental health diagnoses, postpartum depression, and other drivers of significant psychological distress. Having an integrated approach allowed us to respond to cases comprehensively.

The flexibility and collaboration built into the response structure allowed team leaders to adapt the service model to emerging needs identified by team members or newcomers themselves. The changes described between the four phases of the response (i.e., the inclusion of a women's clinic, the use of group interventions for mental health rather than solely individual service provision, etc.) were informed and made possible by a response structure that included frequent feedback loops and opportunities for interdisciplinary communication.

Sharing information using one platform supported this iterative adaptation and was one of the strengths of this model. A centralized case management database was used to collect and manage health response data from the Arrival Health and Safety Check, specialty referrals, follow-up appointments for care coordination, immunization data for vaccine clinics, and more. Concurrent evaluation of this data, coupled with the structure of our response model, ensured programmatic flexibility was centered around newcomers' needs.

Adapting the health response to meet the unique cultural needs of this population was particularly crucial to provide comprehensive and inclusive care. Offering gender- and language-concordant care, preventing unnecessary and retraumatizing emergency department visits, facilitating connections to care, establishing on-site trauma-informed mental healthcare, and offering culturally sensitive care through the Women's clinic were important achievements. Responsive care elicited robust patient-provider engagements. Newcomers who attended the Women's clinic reported detailed health concerns compared to information shared during the Arrival Health and Safety Check appointments. This programmatic success supports the need to continue building patient-centered care models (30).

### 5.3 Prompt interventions to ensure appropriate levels of care

Although initially hosted by Safe Havens, newcomers, they were resettled to states with acute, complex, and unaddressed health needs. Most Afghans received a basic medical screening upon arrival at the Safe Havens. Goetzman et al. (4) highlight the role of Safe Havens in providing these initial medical screenings and acute medical care for newcomers. Despite these reported diagnoses of significant medical concerns at Safe Havens, very limited medical information arrived at State Refugee Health Programs during the initial phases of OAW. This dearth of health information complicated the health response delivered by states and likely resulted in duplication of services. Based on state feedback, ORR started notifying states of upcoming placements of individuals with medical needs, including people who were pregnant or had serious medical conditions. This synchronized warm hand-off helped states coordinate a more robust care plan with local care providers.

This response highlights the benefits of prompt interventions to address acute health and mental health needs. Previous literature emphasizes the importance of adequately tailored and timely support at logical points of access during initial resettlement. Promptly addressing physical health needs has been shown to be a protective factor in decreasing mental health distress and other health difficulties later in resettlement (16, 31–33).

Given the relative paucity of information on the needs identified or care provided at Safe Havens, a primary goal of this model was to assess arrivals within 48 h of arrival. Although most people (54%) were seen within 2 days of arrival, the median time to intake increased over the response period, from 0 days (same day as arrival) in October to over 4 days by February. This delay over time is largely due to the increase in the pace of arrivals and the limited availability of providers completing the Arrival Health and Safety Checks. Despite these setbacks, most arrivals were seen by providers within a few days of their arrival. This is significant, considering the traditional newcomer screening process typically connects refugees to a healthcare provider at least 30 days after arrival (unless urgent health needs arise).

The findings from this integrated response suggests that providing only an initial medical screening for this population would not have been sufficient. Half of all newcomers screened in Minnesota endorsed at least one health concern upon arrival, and 56% needed a specialty referral. Ensuring continual access to care, whether onsite or offsite facilitated by care coordinators, became imperative for this response, and is an important consideration for future, similar responses. Care coordinators offered essential support for basic, yet crucial healthcare needs such as providing eyeglasses, replacing lost prescriptions, and ensuring access to birth control and ongoing primary care.

Another aim of this response was to prevent unnecessary ED visits. Carrico et al.'s (34) study on the use of ED visits by newly arrived refugees identified 60% of ED visits as preventable. Avoiding urgent care and emergency room visits was thus both a significant motivator and outcome of this model. In an analysis of Afghan pediatric ED visits in Indiana between 2021 and 2022, located right next to a Safe Haven, Ulintz et al.'s (35) retrospective study identifies fever as one of the most commonly presenting symptoms in pediatric ED visits (36%) for Afghan children in the first weeks after initial resettlement. Although only 1.8% of Afghan children in our cohort presented with a fever during intake, it was a recurring concern that was addressed in the onsite walk-in clinic, preventing unnecessary visits to the ED.

Much of the immediate federal response to OAW was directed at Safe Havens. Very early in the response, states across the U.S. received limited guidance on federal benefits and health insurance eligibility for Afghan newcomers. Supplemental funding to states for response efforts was also delayed. During this time, individual states filled a major gap in service provision. This response is emblematic of structural issues across immigration and emergency responses. Federal and state assistance that requires the exhaustion of local resources for their activation may overwhelm smaller local systems and leave them compromised to respond adequately.

There is a need to improve access to health and other social services for all newcomers arriving across the U.S. Accordingly, the scalability and sustainability of this model need to be considered. This model largely depended on volunteer medical providers whose work was concentrated over a short period of 5 months, limiting its case use. Rather than relying on ad-hoc response models, building resilient infrastructure that could meet the needs of all arriving newcomers should be a health policy priority. Many structural solutions have been suggested, including better coordination across federal, state, and local agencies and increased funding, among others (36).

We hope this paper encourages other state public health programs to describe their models in response to OAW. Dissemination of these and similar efforts can inform creative solutions to future and ongoing humanitarian health crises involving displaced communities.

## 6 Limitations

This case study is subject to a few limitations. We were unable to quantify the number of urgent care and ED visits prevented by our response. Additionally, children were not systematically assessed for mental health concerns. They were instead referred to the social support team if parents requested assessment or if providers recommended it and parents consented. Furthermore, the Minnesota response was specific to the strong interdisciplinary and interagency relationships that existed through refugee resettlement pathways and may not be generalizable to all contexts. If not already present, these relationships will need to be built in each local context to ensure success in future response efforts. Future evaluations to capture the qualitative experience of Afghan evacuees and healthcare providers who were part of this model would add valuable perspective on the effectiveness of this approach and could inform future initiatives.

## Data availability statement

The raw data supporting the conclusions of this article will be made available by the authors, without undue reservation.

## Ethics statement

This community case study has reviewed and approved by the Minnesota Department of Health's Institutional Review Board. The studies were conducted in accordance with the local legislation and institutional requirements. Written informed consent to participate in this study was not required from the participants in accordance with the national legislation and the institutional requirements.

## Author contributions

MF: Visualization, Validation, Software, Resources, Project administration, Methodology, Investigation, Formal analysis, Data curation, Conceptualization, Writing – review & editing, Writing – original draft. WC: Writing – review & editing, Writing – original draft, Resources, Project administration, Methodology, Investigation, Data curation, Conceptualization. PS: Writing – review & editing, Writing – original draft, Supervision, Resources, Project administration, Methodology, Investigation, Data curation, Conceptualization. SI: Writing – review & editing, Writing – original draft, Project administration, Methodology, Investigation, Data curation, Conceptualization. ME: Writing – review & editing, Writing – original draft, Project administration, Methodology, Investigation, Data curation, Conceptualization. BH-P: Writing – review & editing, Writing – original draft, Project administration, Methodology, Investigation, Conceptualization. NQ: Writing – review & editing, Writing – original draft, Methodology, Investigation, Conceptualization. RS: Data curation, Writing – review & editing, Writing – original draft, Investigation, Conceptualization. HM: Writing – review & editing, Writing – original draft, Investigation, Data curation, Conceptualization. WS: Supervision, Methodology, Writing – review & editing, Writing – original draft, Investigation, Conceptualization. OA: Writing – review & editing, Writing – original draft, Investigation, Data curation, Conceptualization. CH: Writing – review & editing, Writing – original draft, Investigation, Data curation, Conceptualization. KU: Software, Formal analysis, Methodology, Writing – review & editing, Writing – original draft, Investigation, Data curation, Conceptualization. JK: Validation, Resources, Funding acquisition, Writing – review & editing, Writing – original draft, Supervision, Project administration, Methodology, Investigation, Data curation, Conceptualization. MS: Supervision, Project administration, Methodology, Writing – review & editing, Writing – original draft, Investigation, Data curation, Conceptualization. BM: Resources, Project administration, Funding acquisition, Data curation, Writing – review & editing, Writing – original draft, Supervision, Methodology, Investigation, Conceptualization.
