# Supplementary material for: Welcoming new neighbors: Minnesota's rapid response model to address the urgent health needs of Afghan newcomers, 2021–2022
Source: Front Public Health. 2024 Jun 26;12:1413258. doi: 10.3389/fpubh.2024.1413258 (PMC11233686; doi:10.3389/fpubh.2024.1413258)
Supplement: Supplementary file 1 [file Data_Sheet_1.PDF]

DEPARTMENT OF HEALTH

MOBILE HEALTH INITIATIVE
UNIVERSITY OF MINNESOTA
Driven to Discover®

Updated 1/22/2022

Arrival Health & Safety Check

Provider(s) Name(s): ________________________________

INTAKE Date: _______________________ Location: ☐ Airport ☐ Hotel

NAME ______________________________ DOB: _____________ Language: ☐Pashto ☐Dari/Farsi ☐Both

Suggested script: Welcome to Minnesota! We know you have been through a long and difficult journey. We want to make sure you are as healthy as possible during this hard time and are here to help you with health needs. We will be asking these questions to check on your wellbeing and see if there is anything urgent. Our goal is to help you with your immediate health care needs.

- Do you have your medical and vaccine records with you? ☐Yes ☐No
- *Record all immunization dates below. Or, make copies of vaccine records and TB screening results.*

Vaccine	Date Received		Relevant vaccine Notes
**COVID-19** ☐Pfizer ☐Moderna ☐J&J ☐Other_____	#1___________	#2___________	
Tdap or DTap (circle one)	#1___________	#2___________	
IPV	#1___________	#2___________	
Hepatitis A	#1___________	#2___________	
Hepatitis B	#1___________	#2___________	
Varicella	#1___________	#2___________	**TB Screening Results**<br>IGRA: ☐Pos ☐Neg<br>CXR: ☐Normal ☐Abnormal<br>Class B1 TB (abnormal CXR): ☐Yes ☐No
Influenza	#1___________		
HiB	#1___________	#2___________	
MMR	#1___________	#2___________	
Meningococcal	#1___________	#2___________	
Pneumococcal (PCV13)	#1___________	#2___________	

<u>General Health Concerns</u>

- Do you have any concerns about your health today? ☐Yes ☐No
- Are you feeling sick? ☐Yes ☐No
- *I am going to ask you about specific symptoms and if you are having any of these, please let me know*
  - Have you been having any fevers? ☐Yes ☐No
  - Cough or difficulty breathing? ☐Yes ☐No
  - Runny nose, sore throat, ear pain? ☐Yes ☐No
  - Rashes on your skin, itchy skin or scalp? ☐Yes ☐No
  - Diarrhea, stomach pains, vomiting? ☐Yes ☐No
  - Tooth pain? ☐Yes ☐No
  - Do you have vision problems or need glasses? ☐Yes ☐No
  - Do you have pain in any part of your body? ☐Yes ☐No
  - Other things you haven't told me you think I should know? ☐Yes ☐No

If yes, ____

NAME ______________________________ DOB: _____________

- Have you been told you have a health problem? ☐Yes ☐No
  - If yes, would you feel comfortable telling me what it is? ____________________
  - Are you currently taking medication for it? ☐Yes ☐No
  - Have you been told to take any medicines? ☐Yes ☐No
  - If yes, do you have the medicines? ☐N/A ☐Yes ☐No
  - How many days worth of medicine? ____________________
  - Do you have the bottles with you? *Write the medication names copying the bottles.*
    - ☐Yes ☐No ☐N/A ____

Covid-19 Counseling

Script: Covid infection is rising and we want you and your family to stay safe and healthy. COVID vaccine saves lives and is approved for ages 5 and older and for pregnant women as well.

- If not immunized, offer referral to the hotel Friday Vaccine Clinic
- Counsel on masks and physical distancing
- If patient contracts Covid, patient should contact medical team for help with symptom management or worsening symptoms (ie fever, cough, headache, sore throat, body aches, nausea, vomiting, diarrhea)
- If these RED FLAG symptoms occur, patient needs to see doctor immediately (medical clinic onsite or 911 for after hours):
  - Difficulty Breathing
  - Persistent Pain or Pressure in the Chest
  - New Confusion or Inability to wake or stay awake
  - Pale, gray, or blue-colored skin, lips, or nail beds, depending on skin tone

Family Planning

- Are you pregnant? ☐N/A ☐Unsure ☐Yes ☐No
- Some **couples** have asked about family planning and birth control. Do you have any questions?
- Are you planning to have more children in the next year? ☐Yes ☐No
  - If **yes**, recommend the female patient start taking prenatal vitamins three months prior to conception
  - If **no**, offer primary care follow-up to discuss contraceptive methods. Does the family want to be referred to primary care? ☐Yes ☐No

For Adult Female Patients Only (ages 18 and older): Women's Health Clinic Referral

Script: We have a Women's Health Clinic on Thursdays. The clinic is staffed with **all female** staff (including women interpreters and women providers). We refer all adult females (ages 18 and older) to this clinic for routine women specific health care. This will be a time you can ask any women's health specific questions that you may not be able to address during this group setting with children and males present.

- Is it OK if we refer you to this clinic? ☐N/A ☐Yes ☐No

Updated 1/22/2022

Social Support Screening

Script: This is a difficult situation with many stressors and we are here to help you. I want to ask you a few questions about normal reactions to leaving Afghanistan that people may have.

- Have you felt too sad (jigar khun)? ☐Yes ☐No
- Have you been worrying or thinking too much (esterab)? ☐Yes ☐No
- Does sadness or worrying make it difficult to take care of yourself or your family? ☐Yes ☐No

Note for providers: A positive answer to any of these questions warrants further discussion to inquire about the need for referral to the Social Support Team. If positive answer, please offer the patient a follow-up appointment with the social support team. If negative answer, let them know that they can discuss any of these issues at any doctor's appointment if it should come up in the future.

DEPARTMENT OF HEALTH

MOBILE HEALTH INITIATIVE
UNIVERSITY OF MINNESOTA
Driven to Discover®

Updated 1/22/2022

NAME ___________________________________ DOB: _______________

**HEALTH SCREENING (please write legibly)**	
To be completed **if needed for full health intake**.<br><br>Temperature: __________C/F Weight : _______lb/kg<br>Blood Pressure: _____/_____ Heart Rate: __________<br>Oxygen Saturation: _________%	

Medical History
Please record any past medical issues including labs and radiology with dates performed if known.

____

____

____

____

____

**Medications**	**Allergies**
______________________________________	______________________________________
______________________________________	______________________________________
______________________________________	______________________________________
______________________________________	**Social History (clinically relevant for future providers)**
______________________________________	______________________________________
______________________________________	______________________________________

Physical Exam (leave blank if not done)

GENERAL: ____

HEENT: ____

Cardio: ____

Pulm: ____

Abdomen: ____

Skin: ____

Other (neuro, LAD, mental health, etc.:____

Assessment and Plan

____

____

____

____

____

____

Referral Needed ? ☐Yes ☐No

____

____

____

Medication given or called/faxed to the pharmacy ? ☐Yes ☐No

Note for providers: If giving out medication bottles (ie tylenol, etc). Please have patients write instructions about indications and how to take the medication on a separate piece of paper or on the bottle in their own language.

____

____

Patient tested positive for COVID-19? ☐Yes ☐No

Note for providers: Please notify Blain Mamo blain.mamo@state.mn.us and Anne Semenak, (612)-414-6944, (hotel operations team). Counsel patient on isolation and symptom management.

Would the patient benefit from Comprehensive Medication Management (CMM)? ☐Yes ☐No

We have volunteer pharmacists available to discuss an individualized medication care plan. Recommended for:

- Patients taking several chronic or as needed medications (typical 5 or more)
- Patients with chronic disease states that require intensive medication management (e.g. diabetes, hypertension, etc)
- Patients who are experiencing adverse effects from their medications

Urgent referral or prescriptions?

Note for providers: If urgent referrals or prescriptions are ordered, please send a summary email to Blain Mamo blain.mamo@state.mn.us, case managers and Anne Semenak (reply to medical intake email). In the email include: Patient initials, file number, room number, and the referral or prescription information.

Provider Signature:______________________________
